# Delivery of RNAi-Based Oligonucleotides by Electropermeabilization

**DOI:** 10.3390/ph6040510

**Published:** 2013-04-10

**Authors:** Sophie Chabot, Sandrine Pelofy, Justin Teissié, Muriel Golzio

**Affiliations:** 1 Centre National de la Recherche Scientifique (CNRS), Institut de Pharmacologie et de Biologie Structurale (IPBS) BP 64182, 205 route de Narbonne, Toulouse F-31077, France; E-Mails: sophie.chabot@ipbs.fr (S.C.); sandrine.pelofy@ipbs.fr (S.P.); justin.teissie@ipbs.fr (J.T.); 2 Université Paul Sabatier de Toulouse, IPBS, Toulouse F-31077, France

**Keywords:** electroporation, siRNA, miRNA, LNA, siLNA

## Abstract

For more than a decade, understanding of RNA interference (RNAi) has been a growing field of interest. The potent gene silencing ability that small oligonucleotides have offers new perspectives for cancer therapeutics. One of the present limits is that many biological barriers exist for their efficient delivery into target cells or tissues. Electropermeabilization (EP) is one of the physical methods successfully used to transfer small oligonucleotides into cells or tissues. EP consists in the direct application of calibrated electric pulses to cells or tissues that transiently permeabilize the plasma membranes, allowing efficient *in vitro* and *in vivo* cytoplasmic delivery of exogenous molecules. The present review reports on the type of therapeutic RNAi-based oligonucleotides that can be electrotransferred, the mechanism(s) of their electrotransfer and the technical settings for pre-clinical purposes.

## 1. Introduction

RNA interference (RNAi) is a natural process allowing gene silencing at post-transcriptional level [1]. It offers the possibility of targeting and silencing any pathological protein in a specific way [2]. RNAi is mediated endogenously by microRNAs (miRNAs) [3] and experimentally by small interfering RNAs (siRNAs) or miRNA mimics [4]. Both are small (~22 nt) noncoding RNAs that, once loaded into the RNA-induced silencing complex (RISC), bind to their target messenger RNA (mRNA) impairing its translation. As a result, gene expression is suppressed [5,6].

However, clinical success of RNAi has been hampered by its poor cellular uptake and stability. To overcome these problems, progress has been made to develop new technologies to optimize the chemistry of siRNAs on the one hand, and achieve their effective delivery on the other hand [7]. In fact, their physicochemical characteristics (*i.e*., large molecular weight and anionic charges) prevent passive diffusion across the plasma membrane of most cell types. Thus, delivery methods are required to allow oligonucleotides to enter cells while being biocompatible, safe, and targeted. Delivery is therefore one of the major challenges for the development of RNAi-based therapeutics [8].

Electropermeabilization (EP) is a promising non-viral biophysical method for *in vitro* and *in vivo* delivery of various molecules such as drugs [9] and nucleic acids [10,11]. EP was introduced in 1960s [12] and consists in the direct application of external electric field pulses to permeabilize target cells or tissues. Under calibrated electric conditions, these pulses transiently destabilize the plasma membrane, causing its permeabilization [13]. Since the first report in 2002 [14], numerous publications have demonstrated potency of this technique for siRNA delivery [15,16,17,18,19,20]. Efficiency and convenience of this technique (*i.e*., simplicity of the procedure, low cost and speed) led to its extensive use for both external and internal tissues [21,22]. Moreover, very few side-effects have been reported (mostly superficial burns under poorly controlled conditions), emphasizing the innocuousness of this method for clinical use. To date, several preclinical and clinical studies have shown encouraging results by using hydrophilic cytotoxic drugs or plasmid DNA combined with EP demonstrating antitumor effectiveness [23,24,25,26,27,28].

This article reviews the type of therapeutic RNAi-based oligonucleotides that can be electrotransferred and the associated mechanism(s) of their electrotransfer. Elucidation of the mechanisms involved in RNAi-based oligonucleotides electro-delivery will lead to a better optimization of future treatments and will allow the development of new approaches to EP-based therapy.

## 2. How to Perform Electrotransfer of RNAi-Based Oligonucleotides

The basic instrumentation for EP comprises a pulse generator and specific electrodes. However, the definition of the electrical parameters and the design of the electrodes are crucial steps for efficient and safe electrotransfer into the target cells and tissues.

*Electrical parameters.* Electrical conditions are characterized by physical parameters: electric field intensity (E) and number of electric pulses (N), their duration (T) and frequency (F). The definition of these parameters are essential to achieve effective transfer while preserving cell viability and avoiding unwanted effects on the patient (essentially superficial burns and muscle contractions). Depending on the nature of the molecule to be transferred, there are two types of electrical treatment: electrochemotherapy (ECT) and electrogenetherapy (EGT).

ECT is the combination of a cytotoxic (low molecular weight) drug, such as cisplatin or bleomycin, with electric pulses applied to the tumor. This method uses high electric field intensity (kV/cm) of short duration (microseconds). ECT protocols (8 pulses of 100 µs at 1,300 V/cm; 1 Hz) have been approved in human clinics to treat malignant cutaneous and subcutaneous melanoma [29]. ECT has also been used successfully in veterinary medicine for treatment of feline sarcoma [30], perianal and mast cell tumors in dogs [31,32] and equine sarcoids [33].

The EGT parameters allow the electrotransfer of macromolecules (e.g., nucleic acids) for gene therapy purposes. Compared to ECT, this procedure uses lower field intensity (V/cm) with longer duration (milliseconds) to increase the electrophoretic movement of the electrotransferred macromolecule during the electric pulse. In animal models, EGT has been performed in many different tissues: skeletal [34,35], cardiac muscle [36], liver [37,38], skin [39,40], spleen [41], kidney [42], brain [43], joints [44] and tumor [20]. EGT has also been used successfully in veterinary medicine for the treatment of mast cell tumors in dogs [45]. This procedure is a simple way to obtain an efficient transfer of siRNA both *in vitro* and *in vivo* [20].

Other electrical settings are reported. They consist of combinations of pulses of high voltage and short duration (HV, permeabilizing pulse) [46] followed by low voltage and long duration non-permeabilizing pulses (LV, electrophoretic pulse). Studies performed on mice skeletal muscle showed that the HV-LV pulse sequence leads to an efficient gene transfer, rather similar to what was obtained with the EGT parameters [47].

It is of note that the definition of the electrical parameters is depending on the tissue treated. In fact, tissue electrical response depends on its origin, shape and environment. The type of electrodes used also modify the tissue electrical response.

*Electrodes.* Indeed, the success of the EP technique is linked to the proper distribution of the electric field in the tissue, that is dependent on the type of electrodes used. Numerous electrode configurations have been developed for therapeutic purposes: parallel plate, needle, contact wire, *etc.* [48].

The parallel plate electrodes are the most frequently used for electrotransfer. This consists of placing the electrodes on both sides of the tissue prior to electric pulse delivery [11,49]. This simple design has produced high response rates in animal studies [17,50]. Their limitation is that tissue should fit into the inter-electrode space and that the high field at the point of contact of the electrode with the skin can induce superficial burns if sharp angles are present in their design.

If the parallel plate electrodes have been shown to be well suited for treatment of cutaneous tumors, needle electrodes are more efficient in intraoperative settings, for treating the deepest regions or in large animals [51,52]. Needle electrodes are inserted through the skin allowing deeper penetration of the electric field into the tissue. However, with these electrodes, the electric field is heterogeneous as it is confined to the immediate proximity of the needles. A strong burning of the tissues in contact with the needles was reported. Several configurations are in development, such as linear and circular arrays [51].

The contact wire electrodes have been shown to be very efficient and convenient when large tissue surfaces (several square centimeters) must be treated. They are easy to use at the cutaneous level. Crossed configurations in the field distribution can be easily obtained by changing their orientation on the skin surface [48].

New electrode designs are under development in order to adapt field distribution to the geometry of the tumor, enabling cancer cell permeabilization with minimum tissue damage. These improvements are based on numerical modeling, but the irregular shape of the tumor and the heterogeneity of the surrounding layers render this numerical modeling difficult [53].

## 3. Electrotransfer of RNAi-Based Oligonucleotides and Mechanisms

**Figure 1:**
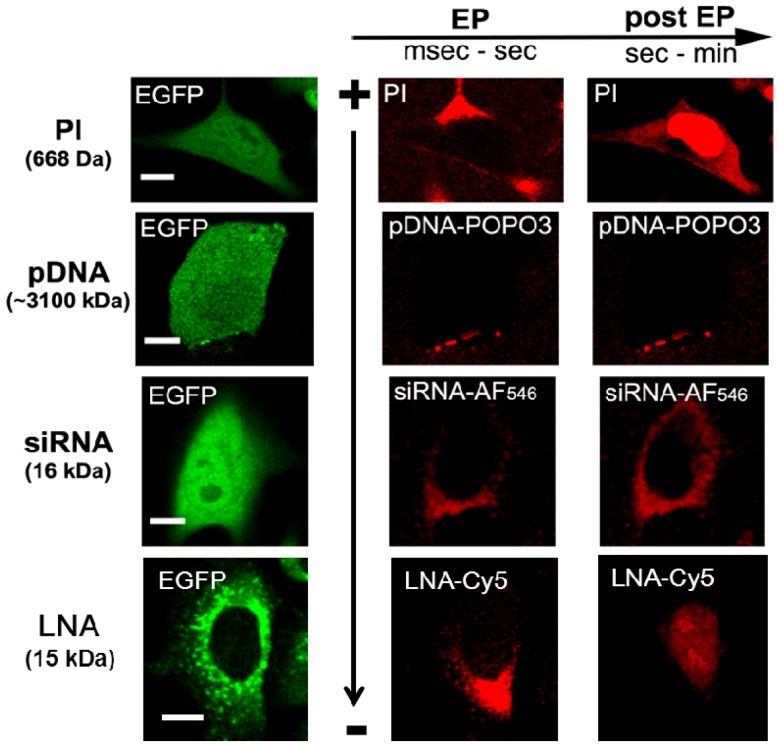
**Mechanisms of electrotransfer of molecules.** Propidium iodide (PI) or small molecules mostly enter the cells by diffusion through both sides of the permeabilized membrane facing the electrodes after the pulse. Plasmid DNA (pDNA), dragged by the electrophoretic forces, interacts with the permeabilized membrane only at the cathode side and remains for a few minutes on the membrane before its translocation into the cytoplasm. siRNA (as well as siLNA) migrates electrophoretically during the pulse through the membrane only at the cathode side, resulting in direct cytosolic localization. LNA-DNA oligomer (LNA) migrates electrophoretically during the pulse through the membrane only at the cathode side, resulting in direct cytosolic localization followed by a rapid nuclear relocalization. The pictures on the left represent stably transfected cells with GFP (adapted from references [50,57]).

EP represents a very attractive delivery method that has led to abundant literature, but only a few reports concern the mechanism of delivery [13]. We showed that electrotransfer of large molecules such as plasmid DNA is a multistep process: electrophoretic migration towards the permeabilized membrane, insertion into the membrane, all within the pulse delivery, followed by a slow translocation across the membrane and migration towards the nucleus [54,55] (Figure 1). Electrotransferred plasmid DNA is not injected into the cytoplasm as observed for small molecules, such as anticancer drugs. Small molecules enter into cells across permeabilized zones of the membrane facing both electrodes. This fast entry occurred mostly by post pulse diffusion process [56] (Figure 1). EP appears also to be well adapted for all kinds of nucleic acids including RNAi-based oligonucleotides [2].

*Small interfering RNA (siRNA).* siRNAs are double-stranded noncoding RNA that once introduced into the cells are loaded into the cytoplasmic RNA-induced silencing complex (RISC). The complex binds the targeted RNA messenger (mRNA) through a base-pairing interaction and leads to its cleavage [58]. Therefore, siRNA offers the possibility to silence the expression of any pathological protein in a specific way. RNAi-based experiments can suffer from a lack of specificity due to silencing of non-targeted genes unless a well-designed sequence is used [59]. Although siRNA *in vitro* efficiency is high, its *in vivo* delivery remains a critical issue for its therapeutic development. A safe approach requests a direct transfer to the cytoplasm to avoid unwanted effects such as interferon response [60].

Using a fluorescently labeled siRNA, we observed by fluorescence microscopy at the single cell level that electrotransferred siRNA was distributed homogeneously throughout the cytoplasm of cultured tumor cells [61]. Thus, upon EP, siRNA had immediate, free access to the cytoplasm allowing its direct interaction with the enzymatic machinery (RISC) and its mRNA target. In these experiments, no electrotransferred siRNA was seen in the nucleus of viable cells. The mechanism of siRNA electrotransfer differs from what is described for plasmid DNA or drugs. siRNA electrotransfer implies electrophoretic movements. However, contrary to plasmid DNA, no complex between the membrane and the siRNA was observed (Figure 1). siRNA electrotransfer led to its direct transfer to the cytoplasm. After pulse application no intracellular diffusion of siRNA occurred although the membrane is still permeabilized for cytotoxic drugs [61].

EP has been used successfully for *in vivo* siRNA electrotransfer in a wide variety of tissues such as muscle [50,62], joint tissue [63], eyes [19], brain [64], kidney [65] and skin [66]. EP has also proved its efficacy in siRNA delivery in tumors [11,67,12]. Similar intracellular localization was observed *in vivo* in mice after intratumor injection of siRNA followed by EP [20]. EP, as compared to other delivery methods such as hydrodynamic transfection, needs a much smaller amount of siRNA to be effective [18,68]. In addition, no immune response was observed with EP, contrary to other delivery technics [69].

*MicroRNA (miRNA)-based oligonucleotides.* Micro-RNAs (miRNAs) are small (~22 nt) non-coding RNAs that post-transcriptionally regulate gene expression by repressing translation or accelerating mRNA decay [5]. miRNAs play crucial roles in the control of critical biological processes, including immune response, cell-cycle control, metabolism, viral replication, stem cell differentiation and human development [70]. miRNA expression or function is significantly altered in many human diseases, including cancer [71,72], cardiovascular diseases [73] and diabetes [74]. Since microRNAs do not require perfect complementarity for target recognition, a single miRNA is able to regulate multiple mRNAs, in contrast to siRNA. Therefore miRNA-based therapy is anticipated to be highly efficacious. Depending on miRNA function and status in disease tissues, there are two approaches to develop miRNA-based therapy: use of antagonists or mimics. The binding of miRNA to its target mRNA by Watson-Crick base-pairing is needed for its biological function [75]. miRNA inhibitors are oligonucleotides that are complementary to their target miRNA and bind to it with high affinity and specificity [76]. Targeting pathways of human diseases with miRNA-based drugs represents a novel and potentially powerful therapeutic approach but again an efficient delivery method is needed [77].

Most of the published reports used systemic delivery [78,79], which implies repetitive high-dose injections, associated with non-specific targeting and toxic side-effects or direct intra-tumor injection alone without any delivery system [80] which is associated with poor tumor uptake and high degradation by tumor nucleases. Rescuing miR-143 expression with *in vivo* electrotransfer of mimic oligonucleotide abrogated prostate cancer growth showing that EP is effective in delivering therapeutic miRNA-based oligonucleotides to tumors *in vivo* [81].

*Chemically modified oligonucleotides.* RNAs are quickly degraded by extra- and intracellular ribonucleases [82,83]. To address this problem and improve RNAi potency and efficacy, approaches based on the introduction of chemical modifications in its sequence have been developed. Therefore, new generations of chemically modified oligonucleotides have been developed [84], including 2′-O-methyl, 2′-methoxyethyl, locked nucleic acids (LNA), and phosphorothioate linkages [85,86]. LNA are nucleotides with a modified backbone. Due to its methylene bridge, the sugar moiety is conformationally locked in an RNA/mimicking C3’-endo/N-type conformation that pre-organizes the base for hybridization [87]. LNA oligonucleotide incorporation into a DNA or RNA oligomer improves the mismatch discrimination compared to unmodified control oligonucleotides [88]. In addition, oligonucleotides containing LNA nucleotides are highly resistant to nuclease degradation and present low toxicity to biological systems [89,90]. The most advanced miRNA-based oligonucleotide is an antagonist LNA specific to the miR-122, which is currently in clinical phase II trial for patients infected with hepatitis C virus [91].

We investigated the electrodelivery of these chemically modified oligonucleotides by fluorescence microscopy. We observed that LNA-DNA oligomer can be efficiently electrotransferred [57]. The number or the position of LNA into the DNA sequence does not interfere with electrotransfer efficiency. The mechanism of LNA-DNA oligomer electrotransfer appears to be closely similar to that just described for siRNA, meaning that LNA-DNA oligomer entry is driven by the electrophoretic forces [57]. LNA-DNA oligomer had a direct access to the cytoplasm and the nucleus where its miRNA target and/or precursor miRNA target, such as pri-, pre-miRNA or miRNA gene, is located. Finally, we demonstrated that electrotransferred LNA/DNA oligomer is biologically functional. EP allowed the homogenous spreading of LNA-DNA oligomer throughout the cytoplasm contrary to lipid-mediated transfection in which LNA-DNA oligomer is shown to be localized in the nuclear periphery in a punctate way, suggesting an endosomal distribution [92,93].

Thus, if chemically modified oligonucleotides appear, in theory, to be promising for RNAi-based therapy, more work for their modifications needs to be performed. Indeed, we showed that modifications of the siRNA with LNA (siLNA) do not interfere with electrotransfer efficiency. However, despite its higher stability and its high electrotransfer efficacy, siLNA was less efficient for eGFP silencing as compared to the electrotransferred unmodified siRNA regardless of the electrical conditions used [94]. Our study highlighted the careful care that is needed when designing chemically-modified oligonucleotides.

## 4. Conclusions

Since the discovery of the RNAi pathway, there has been an explosion of interest in using this technology for clinical applications. Although highly attractive as a therapeutic approach, several hurdles must be overcome to successfully introduce RNAi-based therapies into the clinic. Progress is being made in developing new delivery approaches to provide efficient, safe and localized delivery to cells and tissues. In this context, EP is a promising physical method to target RNAi-based oligonucleotides to the tissue, to facilitate cellular uptake and to give direct access to the intracellular targets. The EP technique is already capable of overcoming many of the delivery problems. Elucidation of the mechanisms involved in RNAi-based oligonucleotides electro-delivery will lead to better planning of future treatments and allow the development of new approaches to EP-based treatments.
